# Equine Metabolic Syndrome Affects Viability, Senescence, and Stress Factors of Equine Adipose-Derived Mesenchymal Stromal Stem Cells: New Insight into EqASCs Isolated from EMS Horses in the Context of Their Aging

**DOI:** 10.1155/2016/4710326

**Published:** 2015-11-22

**Authors:** Krzysztof Marycz, Katarzyna Kornicka, Katarzyna Basinska, Aleksandra Czyrek

**Affiliations:** ^1^Electron Microscopy Laboratory, Wroclaw University of Environmental and Life Sciences, Kozuchowska 5b, 51-631 Wroclaw, Poland; ^2^Wroclaw Research Centre EIT+, 54-066 Wroclaw, Poland

## Abstract

Currently, equine metabolic syndrome (EMS), an endocrine disease linked to insulin resistance, affects an increasing number of horses. However, little is known about the effect of EMS on mesenchymal stem cells that reside in adipose tissue (ASC). Thus it is crucial to evaluate the viability and growth kinetics of these cells, particularly in terms of their application in regenerative medicine. In this study, we investigated the proliferative capacity, morphological features, and accumulation of oxidative stress factors in mesenchymal stem cells isolated from healthy animals (ASC_N_) and horses suffering from EMS (ASC_EMS_). ASC_EMS_ displayed senescent phenotype associated with *β*-galactosidase accumulation, enlarged cell bodies and nuclei, increased apoptosis, and reduced heterochromatin architecture. Moreover, we observed increased amounts of nitric oxide (NO) and reactive oxygen species (ROS) in these cells, accompanied by reduced superoxide dismutase (SOD) activity. We also found in ASC_EMS_ an elevated number of impaired mitochondria, characterized by membrane raptures, disarrayed cristae, and vacuole formation. Our results suggest that the toxic compounds, accumulating in the mitochondria under oxidative stress, lead to alternations in their morphology and may be partially responsible for the senescent phenotype and decreased proliferation potential of ASC_EMS_.

## 1. Introduction

Nowadays, the obesity is an increasing problem in both human and veterinary medicine [1]. The excessive overweight, which accelerates the aging of adipose tissue, becomes one of the serious risk factors of metabolic disorders such as metabolic syndrome or diabetes type II, and evident fact is that lion's share of type 2 diabetic equines are also obese [2]. As recently reported by WHO, diabetes is one of the top ten leading causes of death worldwide in human [3], although these data do not exist in relation to the horse. However, it is well known that EMS are devastating disease that affects more and more horses and causes a myriad of complications that might lead to both patient morbidity and mortality.

Equine metabolic syndrome (EMS) is an endocrine disease, whose pathogenesis is closely linked to insulin resistance (IR), previous or existing laminitis, and global and regional adiposity [4, 5]. The later clinical sign of EMS, that is, “local adiposity,” seems to be extremely important in the context of local inflammation and accelerated aging of adipose tissue taking place inside it. This environment becomes simultaneously the direct tissue milieu for mesenchymal stem cells that reside within adipose tissue. In addition, the action of proinflammatory cytokines, including a broad range of adipokines, such as leptin, resistin, adiponectin, visfatin, and apelin, which are abundantly present in the adipose tissue of EMS horses, cannot remain without physiological relevance for equine adipose-derived mesenchymal stromal stem cells (EqASCs), especially when their clinical application is considered.

Regenerative medicine that involves the application of ASCs becomes a promising tool, increasingly frequently applied in veterinary medicine clinical practice in the treatment of musculoskeletal system disorders. In general, ASCs are classified as unique type of cells characterized by the proliferative potential, ability to self-renew and differentiate* in vitro* into multiple cell lineages [6]. Moreover, in addition to their proliferation and differentiation potential, ASCs exhibit immunomodulatory and immunosuppressive properties, which makes them an even more promising tool in regenerative medicine [7, 8]. Most plausible explanation of ASC proregenerative potential is their paracrine action through secretion of a wide range of growth factors, antiapoptotic and anti-inflammatory factors in membrane-derived vesicles (MVs). MVs are released by ASCs to extracellular space and, in addition to their role as growth factor carriers, they improve cell to cell signalling, which seems to be fundamental in the process of regeneration. Considering the increasing interest in the application of ASCs in equine stem cell-based therapy, there are certain issues that should be addressed prior to their clinical application, particularly in the context of EMS donors. It was shown that the proliferation and differentiation potential of human ASCs (hASCs) derived from obese or insulin resistance patients is negatively correlated with the number and viability of hASCs, and what is more, it positively correlates with cell apoptosis and senescence. Recently, the effect of oxidative stress factors, including reactive oxygen species (ROS) and nitric oxide (NO), has been extensively investigated, because their impact on hASC viability, proliferation, and differentiation had been previously reported. The multipotent character of ASCs was shown to be regulated by the balance between free radicals and antioxidants. Moreover, both ROS and NO were shown to be involved in the initiation of cell apoptosis through the upregulation of the p53 tumor suppressor gene, activation of caspases, chromatin condensation, and DNA fragmentation [9]. In turn, the activity of free radical scavengers, such as superoxide dismutase (SOD), which abolishes the effect of free radicals, may shed a new light on the proliferative status of ASCs derived from EMS donors. The importance of the present study is underlined by the fact that, in human regenerative medicine, MSCs of different origin are considered as potential therapeutic tool for the treatment of diabetes mellitus. Thus, the evaluation of EqASC viability, surface antigen stability, and senescence markers seems to be fully justified, particularly considering their clinical application in EMS treatment.

In the present study, we conducted cellular comparative analysis between EqASCs isolated from healthy individuals and those who suffer from EMS. The fundamental question we were trying to answer was whether EqASCs of EMS individuals were negatively affected by adipose tissue microenvironment, which in consequence might lead to attenuation of their regenerative potential and eventual exclusion from clinical practice, bearing in mind the recommendations of International Society for Cellular Therapy for stem cells applications [10]. To our knowledge, this is the first report that demonstrates that EqASCs isolated from EMS patient are impaired in terms of their surface antigen stability, viability, and clonogenic potential and show increased senescence.

## 2. Materials and Methods

All reagents used in this experiment were purchased from Sigma-Aldrich (Poland), unless indicated otherwise.

### 2.1. Animals Qualifications

All experiments were carried out using protocols approved by the Second Local Ethics Committee (Chelmonskiego 38C, 51-630 Wroclaw, Poland; decision number 84/2012). Procedures were performed under local anaesthesia using 2% lignocaine (Polfa S.A., Poland). All horses were age-matched (mixed sex, 9–14 years; mean ± SD, 11.2 ± 1.7 years) and divided into two groups: EMS group (*n* = 6) and control, healthy horses (*n* = 6). Detailed characterization of animals used in this study is shown in Table 1. Qualification to the experimental groups was performed based on (i) extensive interviews with owners, (ii) measurement of body weight, (iii) estimation of body condition score (BCS) and cresty neck scoring system (CNS), (iv) palpation and visual assessment of the hoof capsule, (v) X-ray examination, (vi) resting insulin levels, (vii) combined glucose-insulin test (CGIT), and (viii) LEP concentration as previously described by Basinska et al. [4].

### 2.2. Cell Collection and Isolation

White, subcutaneous adipose tissue (2 grams) was collected from the horses' tail base, according to the standard surgical procedure and ethical standards, as previously described [4]. After harvesting, specimens were placed in sterile Hank's balanced salt solution (HBSS).

Adipose-derived mesenchymal stem cells were isolated under aseptic conditions following the previously described protocol by Grzesiak et al. [11] and Marycz et al. [12]. Briefly, tissue samples were washed extensively with HBSS supplemented with 1% antibiotic-antimycotic solution (Penicillin/Streptomycin/Amphotericin B) and then cut into small pieces by surgical scissors. The extracellular matrix was digested with collagenase type I (1 mg/mL) for 40 minutes at 37°C and 5% CO_2_. Tissue homogenate was then centrifuged at 1200 ×g for 10 minutes at room temperature (IEC CL31R, Thermo Scientific). The supernatant was discarded and the pellet was resuspended in the culture medium. The cell suspension was then transferred to a culture flask.

### 2.3. Cell Culture

During the experiment, the cells were cultured under aseptic and constant conditions in an incubator (37°C, 5% CO_2_, and 95% humidity). The primary cultures were plated on T-25 culture flasks and cultured in Dulbecco's Modified Eagle's Medium (DMEM) with the F-12 Ham nutrient, 10% Fetal Bovine Serum (FBS), and 1% PSA solution. DMEM containing 4500 mg/L glucose supplemented with 10% FBS and 1% of PSA was used in the secondary cultures. The media were changed every two days and the cells were passaged using trypsin solution (TrypLE Express; Life Technologies, Poland) after reaching 80% confluence. Prior to the experiment, EqASCs were passaged three times.

### 2.4. Immunophenotyping of EqASCs

Isolated cells were characterized by checking the expression of the following surface markers: CD44, CD45, CD79, CD90, and CD105. Prior to immunofluorescence staining, the cells were cultured onto a 24-well plate for 4 days at initial concentration of 1 × 10^4^ cells per well. Subsequently, the cells were fixed using 4% paraformaldehyde (PFA) for 45 minutes at room temperature and washed three times with HBSS. To permeabilize the membranes and block nonspecific protein-protein interactions, the cells were incubated with HBSS containing 0.2% Tween 20 and 5% goat serum. Primary antibody labelling was performed overnight at 4°C with HCAM: CD44 (1 : 100 dilution); 5′-nucleotidase: CD73 (1 : 200 dilution); glycosylphosphatidylinositol-anchored glycoprotein: CD90 (Abcam, 1 : 250 dilution); endoglin: CD105 (1 : 100 dilution); and CD45: protein tyrosine phosphatase, C type receptor (Abcam, 1 : 100 dilution). Goat anti-rabbit IgG conjugated with Atto448 and goat anti-mouse IgG conjugated with Atto448 (1 : 800 dilution) were used as secondary antibodies. To visualize cell nuclei, specimens were counterstained with 4′,6-diamidino-2-phenylindole (DAPI). The cells were observed under an inverted fluorescence microscope (AxioObserverA1, Zeiss) and photographs were acquired using a Power Shot Camera (Cannon). Comparisons of the fluorescent intensity were performed using ImageJ software.

### 2.5. Multipotency Assay

Multipotency of EqASCs was confirmed by osteogenic, chondrogenic, and adipogenic differentiation of cells cultured in StemXVivo kits (R&D Systems) in accordance with manufacturer's instructions. In order to perform the test, the cells were seeded in a 24-well plate at the initial density of 1 × 10^4^ and the media (500 *μ*L/per well) were changed every two days. Prior to the experiments, EqASCs were passaged three times. Cultures expanded in the standard growth medium were used as a control to establish the effectiveness of the differentiation process.

#### 2.5.1. Osteogenic Differentiation

In order to perform osteogenic differentiation, third-passage cells were cultured in media consisting of StemXVivo Chondrogenic Base Media supplemented with 10% of StemXVivo Human Osteogenic Supplement (R&D Systems) and 1% of P/S/A. After 11 days of culture, cells were fixed in 4% PFA for 15 minutes at room temperature. Following three times washing with HBSS cells were stained with 1% Alizarin Red S solution in water for 10 minutes to visualize mineralized matrix. Moreover, we performed RT-PCR to evaluate quantitatively effectiveness of differentiation process. Investigated genes involved are RUNX-2 (runt-related transcription factor 2), BMP-2 (bone morphogenetic protein 2), and COLL-1 (collagen type I). Sequences of primes are listed in Table 2.

#### 2.5.2. Chondrogenic Differentiation

To induce chondrogenic differentiation, third-passage cells were cultured in chondrogenic media consisting of StemXVivo Osteogenic/Adipogenic Base Media (R&D Systems) supplemented with 10% of StemXVivo Chondrogenic Supplement and 1% of P/S/A for 11 days. After that, cells were fixed in 4% PFA, rinsed twice with distilled water for 5 min each, and stained at room temperature for 30 min with Safranin O solution rinsed 5 times with distilled water. We also evaluate quantitatively effectiveness of differentiation process by analysing expression profile of the following genes (RT-PCR): COMP (cartilage oligomeric matrix protein), COLL-2 (collagen type II), and VIM (vimentin). Sequences of primes are listed in Table 2.

#### 2.5.3. Adipogenic Differentiation

To induce adipogenic differentiation, third-passage cells were treated with adipogenic medium for 9 days. Adipogenic medium consists of StemXVivo Osteogenic/Adipogenic Base Media (R&D Systems) supplemented with 10% of StemXVivo Adipogenic Supplement (R&D Systems) and 1% of P/S/A. To visualise formation of intracellular lipid droplets, cells were fixed with 4% PFA, incubated for 5 minutes with 60% isopropanol, and stained with 2% Oil Red O for 10 minutes. Excess stain was removed by washing with HBSS. Next, cells were counterstained with Mayer haematoxylin for 1 minute. After washing 3 times with distilled water, the positive cells containing lipid droplets were stained red.

Cell morphology was then assessed using an inverted epifluorescent microscope (Zeiss, Axio Observer A.1).

### 2.6. Cell Viability, Population Doubling Time (PDT), and Colony Forming Unit-Fibroblasts (CFU-fs) Assay

For assays, the cells were plated in 24-well plates at a concentration of 2*∗*10^4^ per well and inoculated with 0.5 mL volume of culture medium. Cell proliferation rate was evaluated after 24, 96, and 168 hours of the experiment. The number of viable cells in culture was evaluated with resazurin-based assay (TOX-8) in accordance with the manufacturer's protocol. Briefly, culture media were collected and replaced with medium containing 10% of resazurin dye. Then cultures were incubated with a dye in a CO_2_ incubator, 37°C for 2 hours. Absorbance levels of the supernatants were measured spectrophotometrically using a 96-well microplate reader (Spectrostar Nano, BMG Labtech, Germany). Reduction of the dye was evaluated at a wavelength of 600 nm and a reference wavelength of 690 nm.

The number of cells was estimated on the basis of the growth curve calculated in parallel with the cytotoxicity test. To prepare standard curve, cells were seeded at the density of 20^3^, 40^3^, 60^3^, and 80^3^ per well and dye absorbance was measured in relation to certain cells number. Acquired linear trendline equation allowed estimating number of cells after 24, 96, and 168 hours of the experiment in relation to obtained absorbance values. Population doubling time (PDT) was assessed with the support of a population doubling time online calculator [13].

To evaluate the ability of cells to form colonies, EqASCs were seeded in 6-well plates at a density of 10^2^ per well and inoculated into a culture medium. The cells were propagated for 7 days in accordance with the method described previously by Marędziak et al. [14]. After fixation in 4% ice-cold PFA, the cells were stained with pararosaniline. Colonies of more than 50 cells were counted and documented by a Power Shot Camera. The efficiency of colony forming was calculated using the formula presented below, as described elsewhere [15]:

(1) $$\begin{array}{c} \;\text{CFU-fs}\;\left[\;\%\;\right]=\frac{\text{the number of colonies}}{\text{initial cells number}}\times 100\%. \end{array}$$

### 2.7. Morphology, Ultrastructure, and Morphometry

Cell morphology was assessed using an inverted epifluorescent microscope (Zeiss, Axio Observer A.1). Cultures were fixed in 4% ice-cold PFA for 45 minutes at room temperature, washed with HBSS, and permeabilized by 0.2% Tween 20 in HBSS for 15 minutes. After that, the cells were incubated with phalloidin Atto488 in HBSS (Sigma-Aldrich; 1 : 800 dilutions) in the dark for 45 minutes to visualize actin filaments. Cell nuclei were counterstained with DAPI. The results of staining were documented under a microscope using a Power Shot Camera. The morphology of the cells was assessed on the 1st and 7th day of the experiment.

Detailed morphological characteristics of EqASCs were evaluated with a scanning electron microscope (Zeiss EVO LS15). After fixation, the cells were washed in distilled water and dehydrated in a graded ethanol series (concentration from 50 to 100%). Dehydrated cells were sputtered with gold (ScanCoat 6, Oxford), placed in a microscope chamber, and observed using a SE1 detector, at 10 kV of filament's tension.

To perform transmission electron microscopy analysis, the samples were collected and fixed with 2.5% glutaraldehyde for 24 h at 4°C. Then the cells were washed three times with distilled water and incubated for 2 h with 1% osmium tetroxide. The samples were then counterstained with lead citrate and uranyl acetate, dehydrated in a graded series of acetone, and embedded using an Agar Low Viscosity Resin Kit (Agar Scientific Ltd., Essex, UK). The specimens were incubated for 48 h at 60°C to polymerize and subsequently were sectioned into ultrathin slices (70 nm), followed by collecting on copper grids. The observations were carried out using FE-STEM Auriga60 at 20 kV filament tension. Using TEM we evaluated the morphometry (width and length) of mitochondria from both study groups.

The diameter of cell nuclei observed under SEM was calculated based on the representative images. We decided to call the cell nuclei “enlarged” when their diameter was greater than 21 *μ*m as mean EqASCs (isolated from healthy individuals) nuclei diameter was equal to 16.4 *μ*m as previously described by Grzesiak et al. [11]. Subsequently, the percentage of cells with enlarged nuclei occurring in both cultures was calculated. We also calculate, using computer software (ImageJ), the percentage of heterochromatin area in relation to the total nucleus area from photographs obtained from TEM.

### 2.8. Visualisation of Tubulin, Golgi Apparatus, and Endoplasmic Reticulum (ER)

Cell organelles were stained with commercially available kits. Tubulin was stained with a red fluorescent protein, using CellLight Tubulin-RFP, BacMam 2.0 (Life Technologies); Golgi apparatus was labeled with a green fluorescent protein, using CellLight Golgi-GFP, BacMam 2.0 (Life Technologies); and endoplasmic reticulum was stained with a red fluorescent protein using CellLight ER-RFP, BacMam 2.0 (Life Technologies). The differences in fluorescence intensities were measured using ImageJ software. All procedures were performed in accordance with the manufacturer's protocols. Briefly, CellLight reagent was added to the cells in complete culture medium (1 : 80) and mixed gently. Cells were then returned to the culture incubator for 24 hours. After that cells were observed under an inverted epifluorescent microscope using the appropriate filter sets.

### 2.9. Oxidative Stress Factors, Senescence, and p53 Analysis

To visualize senescence-associated *β*-galactosidase, we performed staining using Senescence Cells Histochemical Staining Kit following the manufacturer's protocol. Additionally, the number of viable and dead cells was assessed using Cellstain Double Staining Kit. Moreover, we calculated the percentage of dead cells in culture, based on the staining results. The cells were observed using epifluorescence microscopy and all procedures were conducted in accordance with the manufacturer's instructions.

To evaluate stress levels, the cells were cultured in medium without phenol red for 7 days and the tests were performed on the 1st, 4th, and 7th day. Nitric oxide concentration was assessed with the Griess Reagent Kit (Life Technologies) and superoxide dismutase (SOD) by SOD Assay Kit and the level of reactive oxygen species (ROS) was estimated with a 5-(and-6)-chloromethyl-2′,7′-dichlorodihydrofluorescein diacetate (H2DCF-DA, Life Technologies) solution. All procedures were performed in duplicate in accordance with the manufacturer's protocols. To evaluate extracellular protein levels, the supernatants of cultures were collected on days 1, 4, and 7 and analysed by enzyme-linked immunosorbent assay (ELISA). The level of p53 was determined using Horse Tumor Protein p53 ELISA Kit (MyBioSource).

Fluorescence staining of ROS was performed using Total ROS detection kit for microscopy and flow cytometry (Enzo Life Sciences), whereas mitochondria were stained with a Mito Red fluorescent dye. All procedures were performed in accordance with the manufacturer's protocol.

### 2.10. Quantitative Real-Time Reverse Transcription Polymerase Chain Reaction (qRT-PCR) for p53, p21, BAX, CAS-9, and BCL-2

After 7 days of culture, the cells were rinsed with HBSS and homogenized by TriReagent. Total RNA was isolated using phenol-chloroform method as previously described by Chomczynski and Sacchi [16]. The obtained RNA was then diluted in DEPC-treated water and analyzed in terms of amount and quality using a nanospectrometer (WPA Biowave II). Preparation of DNA-free RNA was performed using DNase I RNase-free Kit (Thermo Scientific). For each reaction, 150 ng of total RNA was used. Complementary DNA (cDNA) was synthesized using a reverse transcriptase Tetro cDNA Synthesis Kit (Bioline). Enzymatic digestion of total RNA and cDNA synthesis were performed in accordance with the manufacturers' instructions using a T100 Thermal Cycler (Bio-Rad).

The qRT-PCR reactions were performed using a CFX Connect Real-Time PCR Detection System (Bio-Rad). Reaction mixture contained 2 *μ*L of cDNA in a total volume of 20 *μ*L using SensiFast SYBR & Fluorescein Kit (Bioline). Primer concentration in each reaction equaled 500 nM; primer sequences used in individual reactions are listed in Table 2. Relative gene expression analysis (Qn) was calculated in relation to the GAPDH housekeeping gene. Moreover we evaluated the ratio between BCL-2 and BAX expression in each group by dividing Qn of BCL-2 by Qn of BAX.

## 3. Results

### 3.1. Immunophenotyping and Multipotency Assay

**Figure 1:**
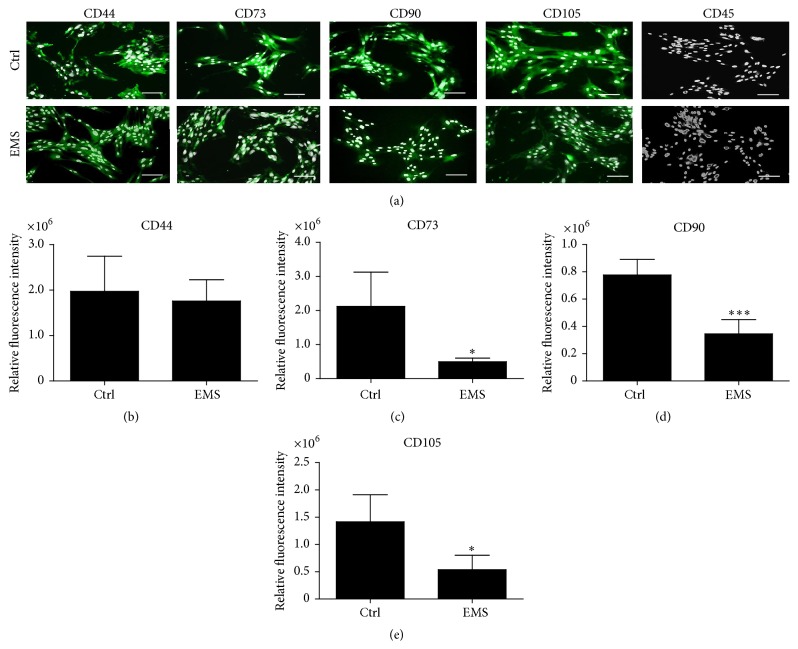
Immunophenotyping of EqASCs. Representative photographs showing the results of EqASC immunophenotyping. (a) The cells expressed the following mesenchymal markers CD44, CD73, CD90, and CD105 and showed no expression of hematopoietic marker CD45. Markers were detected with specific antibodies and visualized with secondary antibodies conjugated with atto-488; nuclei were counterstained with DAPI. Magnification ×100, scale bar: 250 *μ*m. The presence of antigens CD44 (b), CD73 (c), CD90 (d), and CD105 (e) was compared using the relative fluorescent intensity. Results expressed as mean ± SD. ^*∗*^ *p* value < 0.05; ^*∗∗∗*^ *p* value < 0.001.

**Figure 2:**
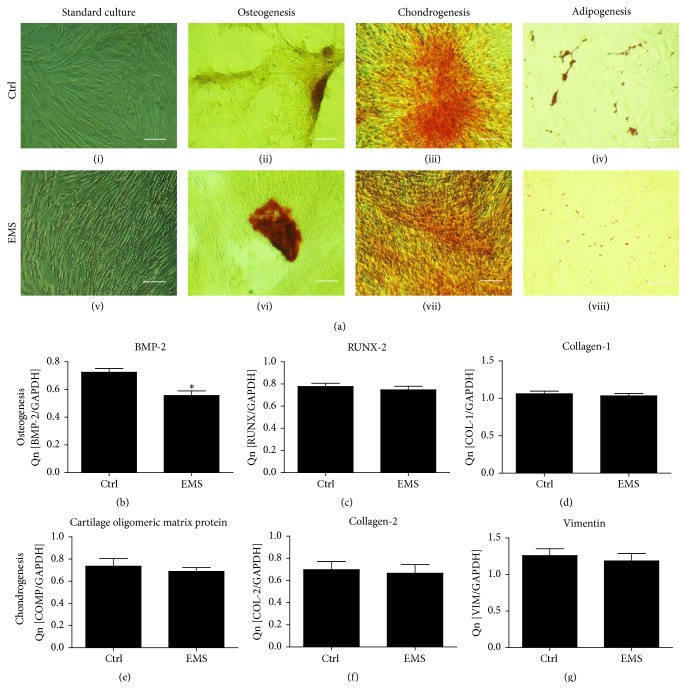
Results of multipotency assay. Morphology of EqASCs cultured under standard, osteogenic, chondrogenic, and adipogenic conditions. Osteogenic differentiation was visualized with Alizarin Red staining on the 11th day; chondrogenic differentiation was confirmed by Safranin O on the 11th day and adipogenesis by Oil Red O staining on the 9th day. Images included in the figure were selected as representative across investigated groups. Magnification ×100, scale bar: 250 *μ*m.

In order to confirm the multipotent character of isolated EqASCs, we performed immunohistochemical staining and multipotency assay. Both cell populations, ASCs_N_ and ASCs_EMS_, expressed the following markers: CD44, CD73, CD90, and CD105. We did not observe the expression of hematopoietic marker CD45 in the investigated groups (Figure 1(a)). Using ImageJ software, we evaluated mean fluorescence intensity and statistical differences between CD73, CD90, and CD105 staining intensities in ASC_EMS_ (Figure 1(b)). Further, multipotent character of the isolated cells was confirmed by the ability of these cells to differentiate into three lineages. EqASCs were able to differentiate into osteogenic, chondrogenic, and adipogenic lineages, which was confirmed by means of appropriate immunohistochemistry (Figure 2). Moreover we performed RT-PCR to evaluate quantitatively effectiveness of differentiation. For osteogenesis, we evaluated expression of BMP-2 (Figure 2(b)), RUNX-2 (Figure 2(c)), and COLL-1 (Figure 2(d)). There was no statistical significance of mRNA levels except BMP-2 whose amount was decreased in ASC_EMS_ in comparison to control group. For chondrogenic differentiation, we evaluate mRNA levels of COMP (Figure 2(e)), COLL-2 (Figure 2(f)), and VIM (Figure 2(g)). Interestingly there were no significance differences in the expression profile between investigated groups.

### 3.2. Cell Proliferation Assay and Morphology Evaluation during Seven-Day Viability Test

**Figure 3:**
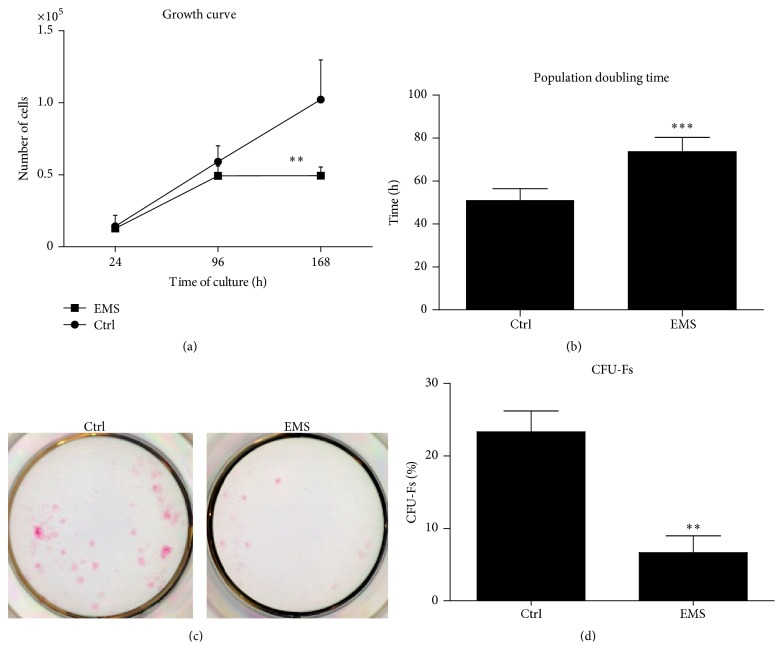
Growth kinetics and clonogenic potential of EqASCs. Mean cells number in both test groups during the seven-day culture period (a) assessed with TOX-8 assay. Time required to double the population of cells expressed in hours (b). Images depicting the results of pararosaniline staining (c). The percentage of colonies consisting of more than 50 cells in the control and EMS group (d). Results expressed as mean ± SD. ^*∗∗*^ *p* value < 0.01; ^*∗∗∗*^ *p* value < 0.001.

Viability characteristics of EqASCs were assessed within seven days of culture. The number of viable cells in culture was evaluated with resazurin-based assay (TOX-8) in accordance with the manufacturer's protocol. The initial cells number on the first day of experiment amounted to 1 × 10^3^ cells/well. During the first 96 hours of culture, we did not observe any significant changes between the ASCs_N_ and ASCs_EMS_ proliferation rate (Figure 3(a)). A significant reduction in the cells number was observed only on day 7 in ASCs_EMS_ in comparison to ASCs_N_ (*p* < 0.01). The estimation of PDT revealed that the ASC_EMS_ cells had markedly extended time of population doubling (*p* < 0.001), as in ASCs_N_ it was 51.12 ± 5.32, while it was 73.75 ± 6.54 in the ASCs_EMS_ group (Figure 3(b)). The greatest number of clonogenic fibroblast precursor cells (CFU-F) was observed in ASCs_N_ (Figure 3(d)). In comparison, CFU-F in ASC_EMS_ was significantly reduced (*p* < 0.01). The quality of colony staining performed with pararosaniline is shown in Figure 3(c). The highest number of colonies is clearly visible in the ASC_N_ sample.

**Figure 4:**
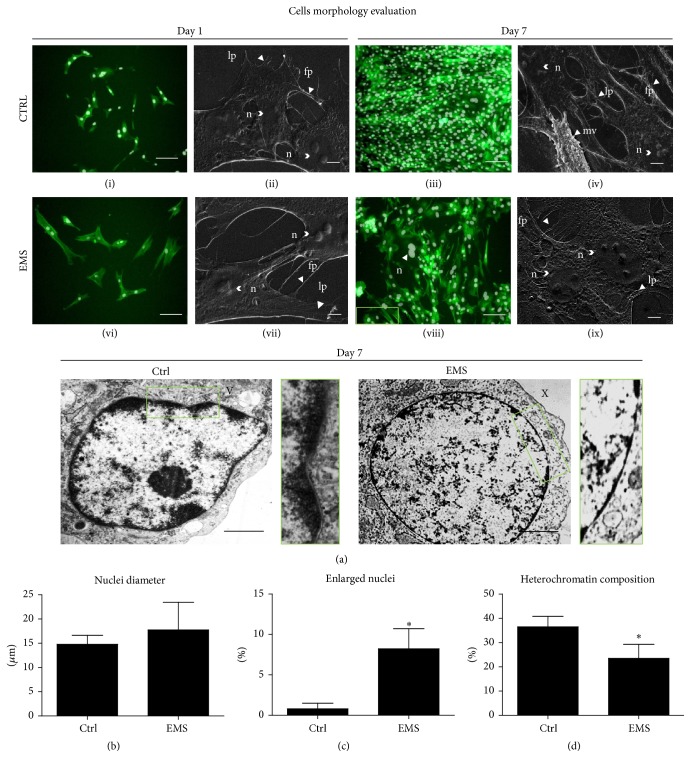
Morphology of EqASCs. The architecture and morphology of the cultures were evaluated on the 1st and 7th day of culture (a). Actin filaments are shown in green and nuclei in white (i, iii, vi, viii). Cell morphology was assessed with a scanning electron microscope at the same time points (ii, iv, vii, ix). ASC_EMS_ showed decreased number of both, filopodia and lamellipodia, as well as decreased production of microvesicles in comparison to control group. Nuclei (n), microvesicles (mv), lamellipodia (lp), and filopodia (fp). TEM images of cell nuclei (V, X). Higher magnification images of boxed regions shown on the right depict heterochromatin underneath the nuclear envelope. EqASC_EMS_ exhibited accelerated breakdown of heterochromatin associated with the inner nuclear membrane. Moreover nuclei of those cells were visibly enlarged. Fluorescent images: magnification ×100, scale bar: 250 *μ*m; SEM images: magnification ×5000, scale bar: 5 *μ*m, TEM magnification ×24000, scale bars: 1 *μ*m. Mean diameter of the nuclei (b), the percentage of enlarged nuclei (c), and the amount of heterochromatin (d). Results expressed as mean ± SD. ^*∗*^ *p* value < 0.05.

Cell morphology was evaluated on the first and last (7th) day of the experiment (Figure 4(a)). Actin filaments were stained green with phalloidin Atto488, whereas cell nuclei were counterstained with DAPI. ASCs_N_ displayed more fibroblast-like, elongated morphology, whereas ASCs_EMS_ were rather flat and no longer of bipolar shape. Staining after 168 h revealed multilayer formation by ASC_N_, whereas the colony confluence in ASC_EMS_ reached approximately 70%. The number of cells in this group decreased, which resulted in a loss of network of intercellular connections. SEM analysis showed that cytoskeletal projections tended to be reduced and shortened in ASC_EMS_. In contrast, ASCs_N_ developed long structures known as lamellipodia and filopodia, which connected adjacent cells. The production of membrane derived microvesicles (MVs) was more robust in ASC_N_ in comparison to ASC_EMS_. Moreover, we observed a high number of enlarged nuclei in ASC_EMS_ (“enlarged” means diameter was greater than 21 *μ*m). EqASC_EMS_ cell nuclei imaged with TEM were characterized by the loss of heterochromatin and its disorganization. Interestingly, EqASC_EMS_ showed reduced heterochromatin architecture at the nuclear periphery.

Using SEM imaging, we estimated mean nuclei diameters in both ASC_N_ and ASC_EMS_ (Figure 4(b)). The nuclei diameters were increased in ASCs_EMS_, although the difference was not statistically significant in comparison to ASCs_N_. Secondly, we calculated the percentage of enlarged nuclei in both cultures (Figure 4(c)). Their number was significantly higher in ASCs_EMS_ (*p* < 0.05). In addition, the architecture of heterochromatin was significantly underdeveloped in ASC_EMS_.

**Figure 5:**
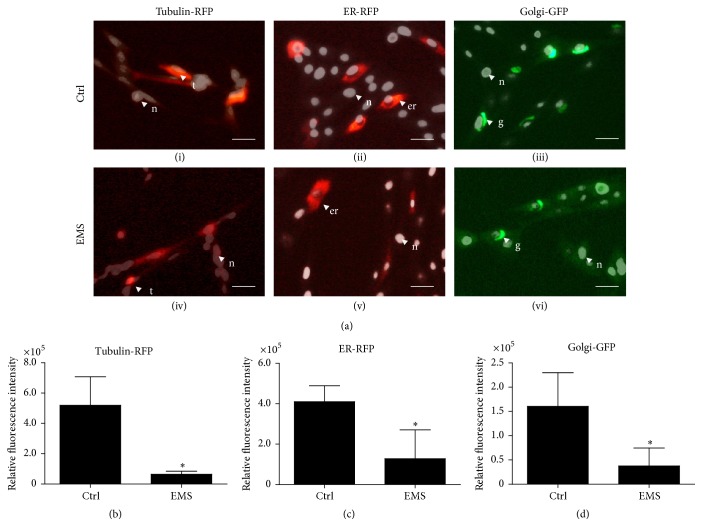
Organelles of EqASCs. Cellular composition of EqASC_N_ and EqASC_EMS_ (a) observed under epifluorescent microscope. Morphological features are indicated with the following abbreviations: n: nucleus; t: tubulin; er: endoplasmic reticulum; g: Golgi apparatus. Images show a qualitative evaluation of the composition of cells in the cultures (a). Magnification ×100, scale bar: 100 *μ*m. Evaluation of the quantitative difference using the relative fluorescent intensity for tubulin-RFP (b), ER-RFP (c), and Golgi apparatus (d). Results expressed as mean ± SD. ^*∗*^ *p* value < 0.05.

Cell organelles were stained with CellLight commercially available kits. After that cells were observed under an inverted epifluorescent microscope using the appropriate filter sets. Staining of particular organelles demonstrated differences in the cellular composition between both study groups (Figure 5(a)). The more intense fluorescence signal for tubulin (red fluorescence), endoplasmic reticulum (red fluorescence), and Golgi apparatus (green fluorescence) was observed in the ASC_N_ group (*p* < 0.05), which was confirmed by ImageJ software analysis (Figures 5(b), 5(c), and 5(d)). The decreased activity of ER and Golgi, as well as tubulin production, may be partially responsible for the reduced metabolic and proliferative activity of ASC_EMS_.

### 3.3. Evaluation of Senescence Markers

**Figure 6:**
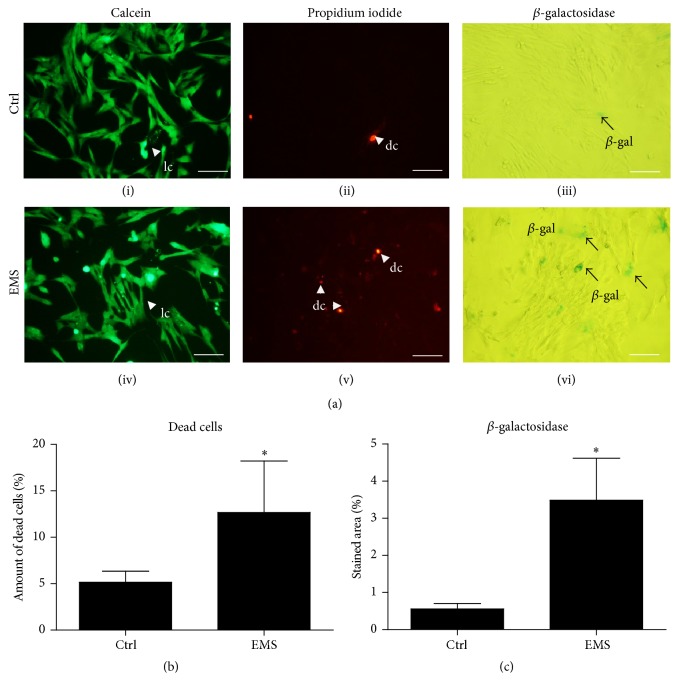
Evaluation of apoptosis and senescence in EqASC_N_ and EqASC_EMS_. Assessment of apoptosis and senescence (a). Pictures showing the results of calcein: live cells (i, iv); propidium iodide: dead cells (ii, v); and *β*-galactosidase: senescence cells (iii, vi) staining. Percentage of dead cells in the cultures was evaluated based on the calcein/propidium iodide staining (b). Differences in the accumulation of *β*-galactosidase were determined based on the percentage of stained area (c). lc: live cell, dc: dead cell, and *β*-gal: *β*-galactosidase. Magnification ×100, scale bar: 250 *μ*m. Results expressed as mean ± SD. ^*∗*^ *p* value < 0.05.

To determine whether the decrease in cell growth results from the level of apoptosis, we evaluated the percentage of dead cells in the culture using calcein and propidium iodide (Figure 6(a)). Quantification of the image data (Figure 6(b)) showed a significant increase (*p* < 0.05) in the number of ASCs_EMS_ dead cells (12.7 ± 5.6%) in comparison to ASCs_N_ (5.2 ± 1.2). Results of *β*-galactosidase staining are shown in Figure 6(a). Moreover we calculated stained area with computer software (Figure 6(c)). These data showed an increase in *β*-gal accumulation in ASCs_EMS_ in comparison to ASC_N_.

### 3.4. Oxidative Stress Factor Analysis and Extracellular p53 Secretion

**Figure 7:**
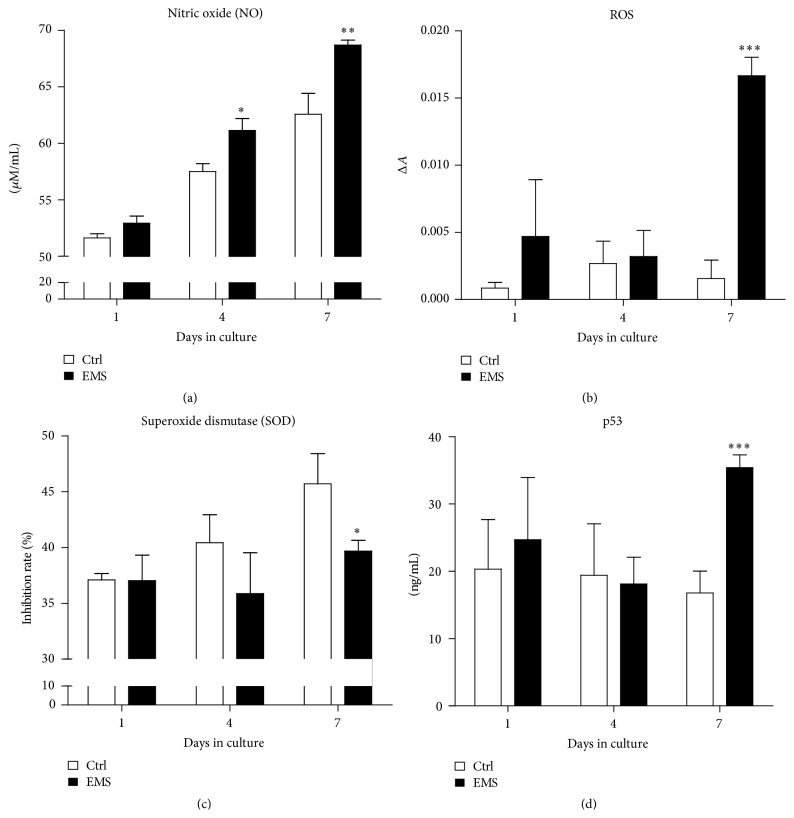
Oxidative stress factors and p53 levels. To evaluate oxidative stress in the culture supernatants, we assessed the levels of nitric oxide (a), reactive oxygen species (b), and superoxide dismutase (c) using commercially available kits on days 1, 4, and 7 of culture. p53 protein level was determined with ELISA (d). Results expressed as mean ± SD. ^*∗*^ *p* value < 0.05, ^*∗∗*^ *p* value < 0.01, and ^*∗∗∗*^ *p* value < 0.001.

During seven days of culture, we recorded a progressively increasing amount of NO in both cultures using Griess reagent. Although on days 4 and 7, we observed a significantly higher accumulation of NO in ASCs_EMS_ compared to ASCs_N_ (*p* < 0.05 and *p* < 0.01, resp.) (Figure 7(a)). ROS level assessed with H2DCFDA in ASC_EMS_ was significantly higher on day 7 (Figure 7(b)). The activity of SOD was increased in ASCs_N_ on day 7, while the activity of SOD was significantly reduced in ASCs_EMS_ (*p* < 0.05) in comparison to the control group (Figure 7(c)). Extracellular signal for p53 was significantly elevated only in ASC_EMS_ on the seventh day of culture (Figure 7(d)).

### 3.5. Imaging of ROS and Mitochondria

**Figure 8:**
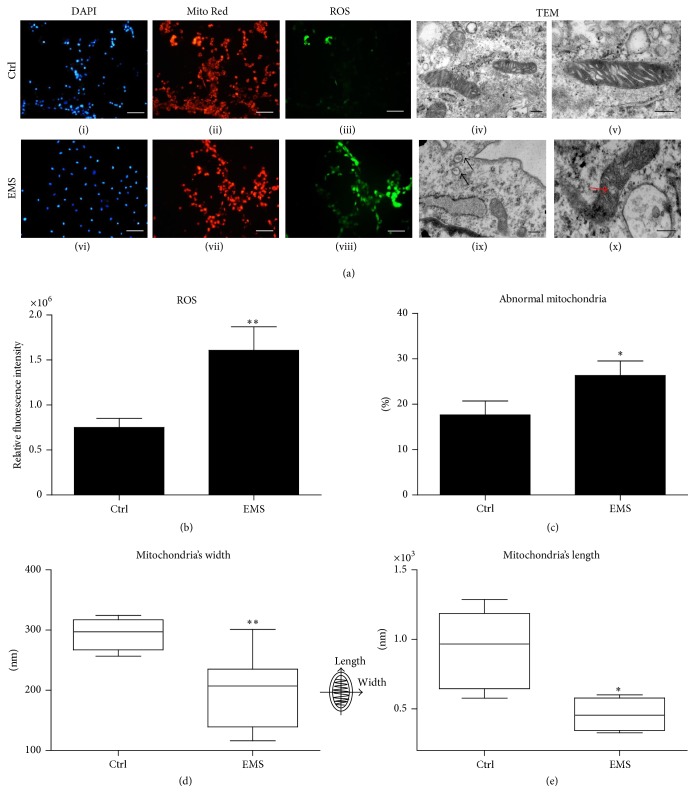
Mitochondria and reactive oxygen species in EqASCs. Images showing DAPI (i, vi), Mito Red (ii, vii), and ROS staining (iii, viii). Magnification ×100, scale bar: 250 *μ*m. Analysis of mitochondrial morphology by a transmission electron microscope (iv, v, ix, x). Representative electron micrographs showing alternations in mitochondrial morphology in EqASC_EMS_. Small structures with disarrayed cristae are indicated with black arrows; membrane raptures and vacuole formation are indicated with red arrows. Quantitative assessment of ROS by the relative fluorescent intensity (b). Percentage of abnormal mitochondria (c); the width (d); and length (e) of mitochondria. Fluorescent images: magnification ×100, scale bar: 250 *μ*m; TEM magnification ×120000, scale bar: 200 *μ*m. Results expressed as mean ± SD. ^*∗*^ *p* value < 0.05; ^*∗∗*^ *p* value < 0.01.

After fluorescence staining of ROS using Total ROS detection kit for microscopy and flow cytometry and mitochondria with a Mito Red fluorescent dye, cells were observed under epifluorescent microscope. Imaging (Figure 8(a)) revealed that the ROS levels were increased in ASC_EMS_. When comparing ROS images with Mito Red staining, it is apparent that most of the ROS was derived from mitochondria. We also evaluated the intensity of ROS fluorescence with ImageJ software. These calculations confirmed the increased production of ROS in ASC_EMS_ (Figure 8(b)). To determine if EMS can affect mitochondrial structure in ASCs, transmission electron microscopy (TEM) analysis was carried out. Representative images are shown in Figure 8(a). Majority of the mitochondria in ASC_N_ presented a typical, bean-shaped structure with numerous cristae and intact membranes. On the contrary, we observed many alternations in ASC_EMS_ mitochondria. They were characterized by a small shape, disarrayed cristae, and membrane raptures. Moreover, vacuoles were present in these mitochondria. Next we calculated the percentage of abnormal mitochondria in ASC_N_ and ASC_EMS_ (Figure 8(c)). Furthermore, we evaluated the morphometry of mitochondria in the study groups. Both the width (Figure 8(d)) and length (Figure 8(e)) of the mitochondria were decreased in ASC_EMS_.

### 3.6. EqASC mRNA Levels of p21, p53, BAX, CAS-9, and BCL-2/BAX Ratio

**Figure 9:**
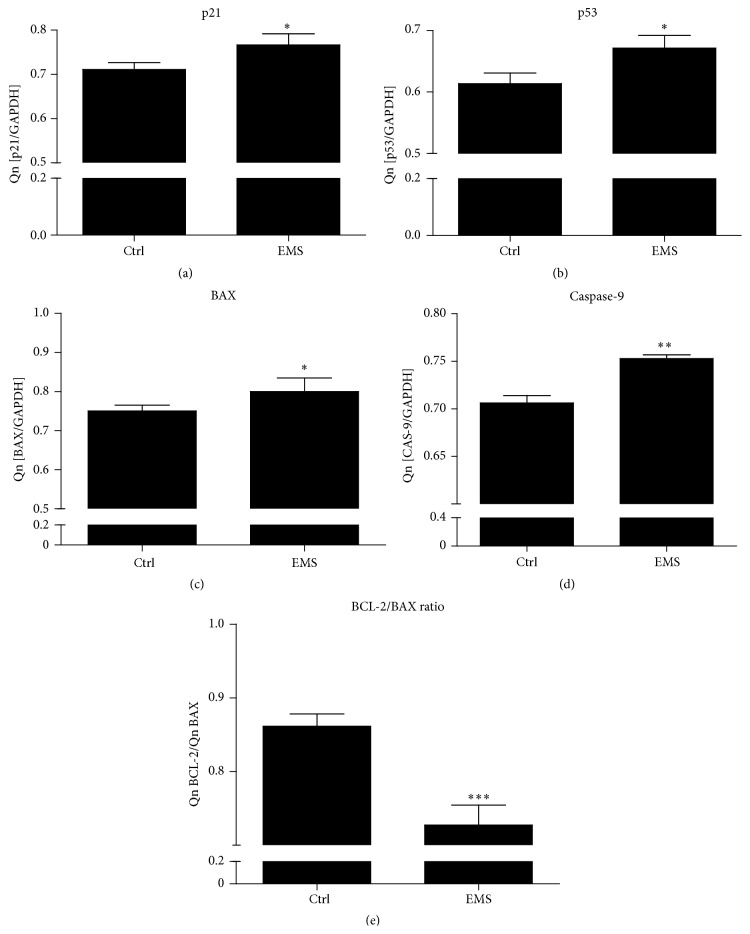
EqASC mRNA levels of p21, p53, BAX, caspase-9, and the BCL-2/BAX ratio. Gene expression in the EqASC culture. The upregulation of apoptotic-related genes observed in ASC_EMS_ results in decreased proliferation potential and senescence phenotype. BAX and CAS-9 increased expression suggest that those cells undergo apoptosis via mitochondrial pathway. mRNA levels of p21 (a), p53 (b), BAX (c), and caspase-9 (d). The ratio between BCL-2 and BAX expression in each group estimated by dividing Qn of BCL-2 by Qn of BAX (e). Results expressed as mean ± SD. ^*∗*^ *p* value < 0.05; ^*∗∗*^ *p* value < 0.01.

Quantitative analysis of p21 (Figure 9(a)) and p53 (Figure 9(b)) mRNA levels using RT-PCR revealed significantly (*p* < 0.05) higher amount of both transcripts in ASCs_EMS_. Moreover, expression of both BAX and caspase-9 mRNA was significantly higher in ASC_EMS_ (*p* < 0.05 and *p* < 0.01, resp.). Moreover the ratio of BCL-2/BAX expression was significantly lower (*p* < 0.001) in ASC_EMS_ making those cells more susceptible for apoptosis (Figure 9(e)).

## 4. Discussion

Obesity has become one of the principal causative risk factors in the development of EMS and it concerns a rapidly growing number of horses all over the world. As recently reported by The World Horse Welfare organization, only in the United Kingdom, obesity concerns 50% of the population of pleasure riding horses [17, 18]. Moreover adipose tissue (AT) is a physiologically complex organ, because it exhibits high metabolic and endocrine activity and directly affects other organ functions. In addition to its energy storage function, AT produces a number of proinflammatory cytokines and adipokines and accumulates oxidative stress factors (OS), which may play a fundamental role in the pathogenesis of EMS. Moreover, OS accumulated in the adipose tissue may affect the proregenerative potential of adipose-derived mesenchymal stromal stem cells and, in consequence, induce aging in this population of cells. Bearing in mind that ASCs are increasingly frequently applied in veterinary regenerative medicine, it is important to understand the effect of oxidative stress on these cells' viability, antigen stability, and finally the senescence.

In the current research we have found that ASCs_EMS_ compared to the control ASCs_N_ exhibited significantly lower proliferative potential, extended population doubling time, and reduced clonogenic potential. Furthermore, the evaluation of surface antigen activity in both investigated groups revealed a substantial reduction of CD90 (Thy-1 cell surface antigen), CD105 (endoglin), and CD73 expression in ASCs_EMS_. This indicates that metabolic syndrome has a negative impact on ASC surface marker expression, which may be partially responsible for disturbances in the proliferative activity of ASCs. To our knowledge, this is the first report that demonstrates dysfunctional expression of surface antigens in ASC_EMS_. The loss of multipotency by ASCs_EMS_ can be a serious limitation, especially when clinical application of these cells in cellular therapy is considered. Our data are partially consistent with the findings of Kočí and colleagues, who demonstrated downregulation of CD105 expression in human ASCs from diabetic patients [19]. Moreover, the study on the diet-induced obese mice showed decreased endoglin (CD105) expression, which altered the properties of adult stem cells [20]. However, we did not observe differences (mRNA levels assessment by RT-PCR) in the effectiveness of osteogenesis and chondrogenesis between investigated groups. This may have crucial implications while considering use of ASC_EMS_ in cellular therapy.

The impaired proliferative activity, elevated senescence, and apoptosis observed in ASCs_EMS_ might be directly linked to oxidative stress (OS). In the current research, we have found that ASCs_EMS_ are more prone to apoptosis (propidium iodide staining) and accumulate increased amounts of senescence-associated *β*-galactosidase, showing in parallel elevated ROS and NO levels. Moreover, ASC_EMS_ displayed typical characteristics of prematurely senescent cells, including enlarged nucleus and widely spread cellular morphology, known as the so-called fried egg shape [21]. Ultrastructural analysis of the nuclei revealed DNA disorganization and decreased quantity of heterochromatin in ASC_EMS_. This phenomenon has been recently shown by Zhang et al. [22] to be involved in premature aging of cells, causing DNA instability and deregulation of gene expression, which results in both retarded cell growth and accelerated cellular senescence, as evidenced by *β*-gal staining* in vitro*.

Increased production of ROS and the resulting cellular oxidative stress have been identified as a major factor contributing to aging [23]. Reduction of molecular oxygen through the electron leakage in the electron transport chain leads to the generation of highly reactive ROS intermediates, such as superoxide anion, hydrogen peroxide, and other minor species [24]. Hence, the imbalance between the excess ROS production and antioxidant capacity would trigger deleterious cellular defects resulting in cell death. In our study, we observed that EMS had a negative impact on the mitochondria in ASCs. Elevated production of ROS in ASCs_EMS_ might be caused,* inter alia*, by a higher systemic leptin concentration in EMS horses. This adipokine, in addition to acting on the limbic system by stimulating dopamine uptake, can directly induce ROS production and, consequently, generate OS. The mechanism explaining this phenomenon can be the mitochondrial and peroxisomal oxidation of fatty acids, which can produce ROS in oxidation reactions. The second possibility may be an overconsumption of oxygen, which generates free radicals in the mitochondrial respiratory chain. ASC_N_ mitochondria in TEM analysis had a bean-shape structure with many transversely oriented cristae enveloped by intact outer membrane. In contrast, numerous morphological alternations were observed in ASC_EMS_, including decrease in size, disarrayed cristae, and decreased electron density. In addition, we observed the formation of vacuoles and increased membrane raptures within mitochondria. These ultrastructural defects had been correlated with mitochondrial metabolic disorders and cell death [25, 26]. Our data are consistent with the results of Wang et al. [27], who observed mitochondrial dysfunction and apoptosis in the cumulus cells of type I diabetes mice. Moreover, taking into consideration the increased amount of p21 transcript in ASC_EMS_, it is likely that those cells undergo apoptosis via the mitochondrial pathway more frequently than ASC_N_. Mitochondria are a major generator of ROS; therefore, we performed fluorescence staining and found increased ROS production in ASC_EMS_, which resulted in mtDNA mutations, as without histones and DNA repair system they are more susceptible to free radical damage. Simultaneously with these observations, we also noticed a decreased antioxidative protection originating from the SOD activity, which additionally suggested an impaired balance between free radicals and antioxidative protection. The data obtained are in good agreement with our previous findings as well as the results of Stolzing et al. [28], who showed a high correlation between OS, apoptosis, and senescence; however, the study was conducted in human ASCs. Further, we observed upregulation of both p21 and p53 genes, which are well known as genes responsible for the induction of apoptosis. Moreover, it has been recently reported that the long-term activation of the p21 checkpoint gene (CDKN1A) induced mitochondrial dysfunction and production of reactive oxygen species (ROS) through signalling pathway involving GADD45-MAPK14 (p38MAPK)-GRB2-TGFBR2-TGFb [29]. This is consistent with our observation that the significant mitochondrial dysfunction in ASCs_EMS_ was associated with simultaneous ROS activity. It is worth noting that the p53 protein level in the intercellular space was elevated in the ASC_EMS_-derived supernatants compared to ASCs_N_. This suggests that the upregulated p53 and p21 genes are both responsible for the impaired proliferative potential of ASCs_EMS_ and their apoptotic susceptibility. Moreover we observed upregulation of both BAX and caspase-9 in ASC_EMS_. The intrinsic apoptotic pathway is activated by cellular stress and it is regulated primarily at the level of mitochondria by the BCL-2 family proteins. One of BCL-2 family member proteins is BAX which targets mitochondrial membranes and increases opening of the mitochondrial voltage-dependent anion channel (VDAC), which leads to the loss in membrane potential and the release of cytochrome c. Once cytochrome c is released to cytosol, it activates caspase by binding to Apaf-1 and inducing it to associate with pro-caspase-9, thereby triggering caspase-9. Following that, the proteolytic cascade that culminates in apoptosis is initiated. Moreover, the expression of this gene is regulated by p53 and has been shown to be involved in p53-mediated apoptosis [30]. In ASC_EMS_, we observed higher amount of BAX, caspase-9, and p53 mRNA in comparison to control group. Taking into consideration facts mentioned above and our own results, it is likely that decrease in proliferation rate of ASC_EMS_ is the result of accumulating ROS and disruption of mitochondria structure. Moreover, those cells seem to undergo apoptosis via p53-Bax mitochondrial pathway. We also evaluated BCL-2/BAX ratio which determines cells survival or death. It is well known that BCL-2 prevents activation of caspase cascade, blocks loss of mitochondrial membrane potential, and also inhibits release of cytochrome c from mitochondria [31]. In our study we observed that BCL-2/BAX ratio was significantly decreased in ASC_EMS_, which affected cell survival by inducing mitochondrial-apoptotic pathway mechanism and increasing cell death rate.

The capacity of p53 to directly activate BAX to permeabilize mitochondria permits an uninterrupted pathway leading from DNA damage and/or ROS accumulation to the mitochondrial release of cytochrome c, caspase activation, and apoptosis. Many studies have demonstrated the role of oxidative stress in the pathogenesis of diabetes. Increased formation of ROS in diabetes can possibly be related to an increase in glucose concentrations in plasma and tissues [32, 33], and it may play a role in the pathogenesis of diabetic nephropathy. Thus, treating ASC_EMS_ with antioxidants before their clinical application to improve mitochondrial function appears to be justified.

## Figures and Tables

**Table 1:** Criteria for dividing the horses into the experimental and control group.

Group	O (number)	Sex	Main clinical parameters					
			Bw	BCS	CNS	Fasting insulin	LEP	CGIT : GLU in 45 min
			(Kg)	(1–9)	(1–5)	(mU/mL)	(ng/mL)	(mg/dL)
Healthy horse	1	f	610	6	1	7	3,21	74/p
	2	f	644	7	2	12	4,12	69/p
	3	f	627	7	2	9	2,87	71/p
	4	m	609	6	1	8	1,86	89/p
	5	m	649	7	2	14	3,56	80/p
	6	m	639	6	2	13	2,91	74/p

Mean ± SD			629,7 ± 15,7	6,5 ± 0,5	1,7 ± 0,5	10,5 ± 2,6	3,1 ± 0,7	76,2 ± 6,7

Horse with EMS	1	f	710	8	3	83	4,89	138/p
	2	f	726	9	3	67	5,19	141/p
	3	f	760	9	4	98	9,12	140/p
	4	m	709	8	3	73	8,49	136/p
	5	m	716	8	4	69	7,27	134/p
	6	m	746	9	4	82	8,36	146/p

Mean ± SD			727,8 ± 19,1	8,5 ± 0,5	3,5 ± 0,5	78,7 ± 10,5	7,2 ± 1,6	139,2 ± 3,8

f: female, m: male, BW: body weight, BCS: body condition score, CNS: cresty neck score, CGIT: combined glucose-insulin test, SD: standard deviation, LEP: leptin, GLU: glucose, p: positive test results, and n: negative test results.

**Table 2:** Sequences of primers used in qPCR.

Gene	Primer	Sequence 5′-3′	Amplicon length	Accession number	Annealing temperature
GAPDH	F:	GATGCCCCAATGTTTGTGA	250	NM_001163856.1	61,7°C
	R:	AAGCAGGGATGATGTTCTGG			

p53	F:	TACTCCCCTGCCCTCAACAA	252	U37120.1	57,6°C
	R:	AGGAATCAGGGCCTTGAGGA			

p21	F:	GAAGAGAAACCCCCAGCTCC	241	XM_003365840.2	52,1°C
	R:	TGACTGCATCAAACCCCACA			

BAX	F:	TTCCGACGGCAACTTCAACT	150	XM_005607505.1	60,5°C
	R:	GGTGACCCAAAGTCGGAGAG			

CAS-9	F:	TCCTACTCCACCTTCCCAGG	150	XM_005607505.1	60,8°C
	R:	CTCCGAAACAGCGTGAGCTA			

BCL-2	F:	TTCTTTGAGTTCGGTGGGGT	164	XM_001490436.2	65,0°C
	R:	GGGCCGTACAGTTCCACAA			

BMP-2	F:	CGTCCTGAGCGAGTTCGAGT	249	XM_001493895.4	60,0°C
	R:	CGCCGGGTTGTTTTTCCACT			

RUNX-2	F:	CCAAGTGGCAAGGTTCAACG	165	XM_005603968.1	60,2°C
	R:	TGTCTGTGCCTTCTGGGTTC			

COLL-1	F:	GAAACTATCAATGGTGGTACCAAGT	265	XM_008524258.1	55,0°C
	R:	AGCAGCCATCTACAAGAACAGT			

COMP	F:	AGTGTCGCAAGGATAACTGCGTGA	238	NM_001081856.1	62,6°C
	R:	TCCTGATCTGTGTCCTTCTGGTCA			

COLL-2	F:	ATTCCTGGAGCCAAAGGATCTGCT	148	XM_005611082.1	62,7°C
	R:	TGAAGCCAGCAATACCAGGTTCAC			

VIM	F:	GCAGGATTTCTCTGCCTCTT	203	NM_001243145.1	55,0°C
	R:	TATTGCTGCACCAAGTGTGT			

Sequences, amplicon length, and annealing temperature of the primer sets. GAPDH: glyceraldehyde 3-phosphate dehydrogenase; p53: tumor suppressor p53; p21: cyclin-dependent kinase inhibitor 1A; BAX: BCL-2-associated X protein; BCL-2: B-cell lymphoma 2; CAS-9: caspase-9; BMP-2: bone morphogenetic protein 2; RUNX-2: runt-related transcription factor 2; COLL-1: collagen type I; COMP: cartilage oligomeric matrix protein; COLL-2: collagen type II; VIM: vimentin.
